# A third COVID-19 vaccine dose in kidney transplant recipients induces antibody response to vaccine and Omicron variants but shows limited Ig subclass switching

**DOI:** 10.1128/spectrum.02190-24

**Published:** 2025-01-31

**Authors:** Jenny M. Lee, Jaiprasath Sachithanandham, John S. Lee, Janna R. Shapiro, Maggie Li, Ioannis Sitaris, Stephanie R. Peralta, Camille Wouters, Andrea L. Cox, Dorry L. Segev, Christine M. Durand, Mark Robien, Aaron A. R. Tobian, Andrew H. Karaba, Joel N. Blankson, William A. Werbel, Andrew Pekosz, Sabra L. Klein

**Affiliations:** 1 W. Harry Feinstone Department of Molecular Microbiology and Immunology, Johns Hopkins University Bloomberg School of Public Health, Baltimore, Maryland, USA; 2 Department of Medicine, Johns Hopkins University School of Medicine, Baltimore, Maryland, USA; 3 Department of Surgery, New York University Grossman School of Medicine and NYU Langone Health, New York, New York, USA; 4 National Institute of Allergy and Infectious Diseases, Bethesda, Maryland, USA; 5 Department of Pathology, Johns Hopkins University School of Medicine, Baltimore, Maryland, USA; Children's National Hospital, George Washington University, Washington, DC, USA

**Keywords:** COVID-19, immunocompromised hosts, SARS-CoV-2, IgG isotype, vaccines

## Abstract

Solid organ transplant recipients (SOTRs) suffer more frequent and more severe infections due to their compromised immune responses resulting from immunosuppressive treatments designed to prevent organ rejection. Pharmacological immunosuppression can adversely affect immune responses to vaccination. A cohort of kidney transplant recipients (KTRs) received their third dose of ancestral, monovalent COVID-19 vaccine in the context of a clinical trial and antibody responses to the vaccine strain, as well as two Omicron variants BA.1 and BA.5 were investigated and compared with healthy controls who also received a third dose of mRNA vaccine (HCs). Total IgG and live virus neutralizing antibody titers were reduced in KTRs compared with controls for all variants. KTRs displayed altered IgG subclass switching, with significantly lower IgG3 antibodies. Responses in KTRs were also very heterogeneous, with some individuals showing strong responses but a significant number showing no Omicron-specific neutralizing antibodies. Taken together, immune responses after COVID-19 vaccination in KTRs were not only lower than HCs but highly variable, indicating that simply increasing the number of vaccine doses alone may not be sufficient to provide greater protection in this population. These findings underscore the need for tailored vaccination strategies for immunosuppressed populations, such as KTRs. Alternative formulations and doses of COVID-19 vaccines should be considered for people with severely compromised immune systems, as more frequent vaccinations may not significantly improve the response, especially regarding neutralizing antibodies.

**IMPORTANCE:**

This study addresses the challenges faced by kidney transplant recipients (KTRs) in mounting effective immune responses against COVID-19. By evaluating the antibody responses to a third dose of monovalent mRNA COVID-19 vaccine and its effectiveness against Omicron subvariants (BA.1 and BA.5), this study reveals significant reductions in both binding and neutralizing antibodies in KTRs compared with healthy controls. The research highlights altered IgG subclass switching and heterogeneous responses within the KTR population. Reduced recognition of variants, coupled with differences in IgG subclasses, decreases both the quality and quantity of protective antibodies after vaccination in KTRs. These findings underscore the need for tailored vaccination strategies for immunosuppressed populations, such as KTRs. Alternative formulations and doses of COVID-19 vaccines should be considered for people with severely compromised immune systems, as more frequent vaccinations may not significantly improve the response, especially regarding neutralizing antibodies.

## INTRODUCTION

SARS-CoV-2 vaccines have remarkable efficacy and safety in both clinical trials and real-life practices (1–5). However, early SARS-CoV-2 vaccine trials and studies excluded immunocompromised populations, including individuals with immune dysfunction and those receiving immunosuppressant mediations (6, 7). This exclusion raises concerns regarding the comprehensive understanding of vaccine responses and immunization strategies among immunocompromised individuals. COVID-19 continues to pose a threat to immunocompromised individuals due to their dampened immune responses to SARS-CoV-2 vaccines, causing an increased risk of severe COVID-19 outcomes and breakthrough infection after SARS-CoV-2 vaccination (8–10).

Solid organ transplant recipients (SOTRs) have an elevated risk of severe COVID-19 infection and mortality, mainly due to immunosuppressive medications administered post-transplantation (11–15). Studies illustrate that breakthrough infection is more common among fully vaccinated SOTRs compared with the immunocompetent general population (16, 17). Following the initial two-dose mRNA vaccine series, many SOTRs exhibit reduced humoral and cellular responses against SARS-CoV-2 (18–23). The proportion of non-responders, defined as no detectable anti-SARS-CoV-2 spike protein antibodies, is considered high among SOTRs with a range from 18% to 100% (24). Given the impaired vaccine-induced responses after a two-dose mRNA vaccine series, an additional vaccine dose was authorized for certain immunocompromised individuals (25). Published studies on the third dose mRNA vaccine in SOTRs showed increased total anti-Spike (S) IgG antibodies and neutralizing antibodies compared with the initial dose series, suggesting substantially higher immunogenicity (26–30). Amid the emergence of SARS-CoV-2 variants of concern (VOCs), the administration of booster vaccines became a critical measure to protect individuals against more transmissible variants, such as the Delta and Omicron variants. To date, there are bivalent boosters (2022–23 containing ancestral and BA.5 Spike) and updated monovalent COVID-19 vaccines (2023–24 containing XBB.1.5 spike protein and 2024–25 containing KP.2 or JN.1 spike). These booster vaccines demonstrated significant immunogenicity against VOCs in immunocompetent individuals (31–35).

While previous studies have examined binding and neutralizing responses in the SOTR population, our study focuses on antibody quality—Omicron subvariant-specific antibody and IgG subclass responses—compared with healthy controls (HCs). Specifically, an in-depth analysis of S-binding antibody subtypes and live virus neutralizing antibody responses in kidney transplant recipients (KTRs, *n* = 81) and HCs (*n* = 11) (Table 1) was performed to understand KTR responses to the vaccine and antigenically distinct SARS-CoV-2 variants to determine the effectiveness of a third vaccine dose in this highly vulnerable population.

**TABLE 1:** Baseline characteristics, vaccine platform, and immunosuppressive regimen of kidney transplant recipient (KTR) and healthy control (HC)^*a*^

	Kidney transplant recipient (KTR)	Healthy control (HC)	
	Total (*n* = 81)	Total (*n* = 11)	
Demographics			*P*-value^*d*^
Age, no. (%)			<0.001
Median (IQR)	66 (57,73)	-	
18–49	13 (16)	8 (73)	
50–64	23 (28)	3 (27)	
≥65	45 (56)	0 (0)	
Sex, no. (%)			0.777
Female	26 (32)	4 (36)	
Male	55 (68)	7 (64)	
Race, no. (%)			
White	49 (60)	-	-
Black/African American	24 (30)	-	
Asian	7 (9)	-	
Ethnicity, no. (%)			
Hispanic/Latino	3 (4)	-	-
Non-Hispanic/Latino	79 (96)	-	
Prefer not to answer	0 (0)	-	
COVID-19 and vaccination history			
Prior SARS-CoV-2 infection^*b*^, no. (%)	4 (5)		
Days between 2nd and 3rd doses, median (IQR)	167 (149,177)		
Vaccine manufacturer, no. (%)			
Pfizer-BioNTech (BNT162b2)	59 (73)		
Moderna (mRNA-1273)	22 (27)		
Transplant history			
Years since transplant, median (IQR)	5.4 (2.1,10.5)		
Medical comorbidities, no.(%)			
Diabetes	26 (32)		
HCV infection	4 (5)		
Lung disease	16 (20)		
Cardiovascular disease	72 (89)		
Autoimmune disease	8 (10)		
Transplant immunosuppression			
Baseline immunosuppressant, no. (%)			
Mycophenolate mofetil	55 (68)		
Mycophenolic acid	9 (11)		
High-dose mycophenolate	14 (22)		
Prednisone	75 (93)		
Tacrolimus	74 (91)		
Cyclosporine	5 (6)		
Triple IS, no. (%)^*c*^	57 (70)		

^*a*^ IQR, interquartile range; Triple IS, triple immunosuppression.

^*b*^ By positive prior molecular testing or reactive anti-nucleocapsid antibody at enrollment.

^*c*^ Any combination of three transplant immunosuppressants (triple therapy) with a low-dose cyclosporine, azathioprine, and prednisone immunosuppression regimen at Day 0.

^*d*^ Categorical variables (age category, gender, race, etc.) were compared between KTR cohort and control by chi-square tests.

## MATERIALS AND METHODS

### Study background and design

This study used 81 samples from the COVID-19 Protection After Transplant (CPAT) pilot trial funded by the National Institutes of Health, a single-arm open-label trial of the safety and immunogenicity of a third dose of SARS-CoV-1 mRNA vaccination in kidney transplant recipients who had failed to respond to two prior mRNA vaccinations (NCT04969263) (36). Study participants were primarily male (55/81; 68%) and White (49/81; 60%), with a median age of 66 years (57, 73) (Table 1). Participants had a median of 5.4 years (range, 2.1–10.5) since transplantation. Cardiovascular disease was the major medical comorbidity (72/8; 89%), followed by diabetes (26/81; 32%), lung disease (16/81; 20%), autoimmune disease (8/81; 10%), and hepatitis C virus infection (4/81; 5%). Patients received a third dose of either mRNA-1273 (22/81; 27%) or BNT162b2 (59/81; 73%) after being fully vaccinated with the initial two-dose series of COVID-19 mRNA vaccine series. Most participants were taking prednisone (75/81; 93%), tacrolimus (74/81; 91%), and mycophenolate mofetil (55/81; 68%) as their immunosuppressant treatments. More than half were undergoing triple immunosuppression (triple IS) therapy (57/81; 70%). Serum samples were collected pre-vaccination, 30 days, and 90 days following the third-dose COVID-19 mRNA vaccine (D3). Responses were compared with 11 healthcare workers (HC) who also received a third dose of mRNA vaccine, serving as immunocompetent control population (Table 1).

### Enzyme-linked immunosorbent assays (ELISAs)

Standardized and validated indirect ELISAs were used to measure the titer of Spike and nucleocapsid (N)-specific IgG against the vaccine strain, and Omicron variants of SARS-CoV-2, as previously described (37–41). Recombinant SARS-CoV-2 S (2 μg/mL) produced at Johns Hopkins University or obtained through NCI Serological Sciences Network for COVID-19 were used to coat 96-well ELISA plates (Immulon 4HBX, Thermo Fisher Scientific) and were incubated overnight at 4°C. Plates were washed with phosphate-buffered saline with 0.1% Tween 20 (PBST) wash buffer (Thermo Fisher Scientific), blocked with 3% nonfat milk in PBST, and incubated for 1 h at room temperature (RT). After incubation, the blocking buffer was discarded. Three-fold serially diluted serum samples, monoclonal antibody against the SARS- CoV-2 S protein (positive control; catalog 40150-D001, Sino Biological), and negative control samples were added and incubated for 2 h at RT. Plates were washed with PBST, and horseradish peroxidase (HRP)-conjugated secondary IgG antibody (catalog A18823, Invitrogen, Thermo Fisher Scientific) was added at a 1:5,000 dilution and incubated for 1 h at RT. For characterization of subclass-specific IgG, we used secondary IgG1 antibody (catalog 9054-05, SouthernBiotech) at a 1:4,000 dilution, IgG2 antibody (catalog 9060-05, SouthernBiotech) at a 1:4,000 dilution, IgG3 antibody (catalog 9210-05, SouthernBiotech) at a 1:4,000 dilution, and IgG4 antibody (catalog 9200-05, SouthernBiotech) at a 1:8,000 dilution. Plates were washed with PBST, and reactions were developed by adding SIGMAFAST OPD (o-phenylenediamine dihydrochloride) solution (MilliporeSigma), followed by a 10-min incubation at RT. Reactions were stopped by adding 3 M hydrochloric acid solution (Thermo Fisher Scientific). The optical density (OD) of each plate was read at 490 nm wavelength on a SpectraMax i3 ELISA Plate Reader (BioTek Instruments). Results were expressed as the log10-transformed area under the curve (AUC) generated from the background-subtracted OD values of the 10 threefold serial dilutions, as previously described (40). The limit of detection (LOD) was determined to be half of the lowest measured AUC with a corresponding endpoint titer of at least 20.

### Cells and viruses

Vero-E6-TMPRSS2 cells were obtained from the cell repository of the National Institute of Infectious Diseases, Japan, and were grown in complete media (CM) consisting of Dulbecco’s modified eagle medium (DMEM) containing 10% fetal bovine serum (FBS) (Gibco, Thermo Fisher Scientific), 1 mM glutamine (Invitrogen, Thermo Fisher Scientific), 1 mM sodium pyruvate (Invitrogen, Thermo Fisher Scientific), 100 U/mL penicillin (Invitrogen, Thermo Fisher Scientific), and 100 µg/mL streptomycin (Invitrogen, Thermo Fisher Scientific). Cells were incubated at 37°C in a humidified incubator with 5% CO^_2_^.

SARS-CoV-2 isolates B.1 (hCoV-19/USA/DC-HP00007/2020; B.1, GISAID EPI_ISL_434688), Omicron BA.1 variant (SCV2/USA/MD-HP20874/2021; BA.1.18, GISAID EPI_ISL_7160424), and Omicron BA.5 (hCoV-19/USA/MD-HP32103-PIDCNSQVGY/2022 GISAID EPI_ISL_15013106) were isolated from Vero-E6-TMPRSS2 cells plated in 24-well plates and grown to 75% confluence (42). The CM was removed and replaced with 150 µL of infection media (IM). IM is identical to CM except with an FBS concentration of 2.5%; 150 µL of the virus transport media containing a SARS-CoV-2 positive clinical swab was added to the culture. The cultures were incubated at 37°C for 2 h, the inoculum was aspirated and replaced with 500 µL of IM, and the cells were incubated at 37°C for up to 5 days. When cytopathic effect (CPE) was visible in most of the cells, the IM was collected and stored at –65°C. The presence of SARS-CoV-2 was verified by extracting RNA from the harvested supernatant using the QIAGEN QIAamp Viral RNA extraction kit, and viral RNA was detected using quantitative RT-PCR. SARS-CoV-2 whole genome sequencing was performed on the cell culture viral isolates based on NGS technology. The consensus sequence of the viral isolate did not differ from the sequence derived from the clinical specimen. The infectious viral titer was determined on Vero-TMPRSS2 cells using a 50% tissue culture infectious dose (TCID50) assay (43). Serial 10-fold dilutions of the viral stock were made in IM, and 100 µL of each dilution was then added to the cells in a 96-well plate in hexaplicate. The cells were incubated at 37°C for 4–5 days, fixed with 4% formaldehyde for at least 4 h, and stained with naphthol blue–black overnight. Plates were scored visually for CPE. The TCID50 per mL was determined using the Reed and Muench calculation.

### Microneutralization assay

Plasma nAbs were determined as described for SARS-CoV and modified for SARS-CoV-2 (41, 44). Twofold dilutions of plasma, starting at a 1:20 dilution, were made using IM. Infectious virus was added to the plasma dilutions at a final concentration of 1 × 10^3^ TCID50/mL or 100 TCID50/100 µL. The plasma–virus solution was incubated at room temperature for 1 h, and 100 µL of each dilution was added to 1 well of a 96-well plate of VeroE6-TMPRSS2 cells in hextuplicate. The cells were incubated for 6 h at 37°C with 5% CO_2_. The inoculum was replaced with fresh IM, and the cells were incubated at 37°C with 5% CO_2_ for 3–4 days until CPE is evident in the negative controls. The cells were fixed by the addition of 100 µL of 4% formaldehyde for at least 4 h at room temperature, and then stained with naphthol blue–black overnight. The nAb titer was calculated as the highest serum dilution that eliminated the CPE in 50% of the wells (NT50), and the AUC was calculated using GraphPad Prism.

### Statistical analyses

Statistical calculations were performed using GraphPad Prism 8 (GraphPad Software) and Stata 15 (StataCorp). Data are shown as mean ± 95% CI (CI) except where otherwise indicated. Demographics and clinical characteristics were evaluated with descriptive statistics. Comparison between antibody responses at pre- and post-vaccination and comparison of antibody responses among SARS-CoV-2 VOC were analyzed using one-way repeated measures ANOVA test. A *P* value less than 0.05 was considered significant.

## RESULTS

### KTRs mount lower vaccine-induced serological responses than healthy controls (HCs) after receipt of a third-dose vaccine

**Fig 1:**
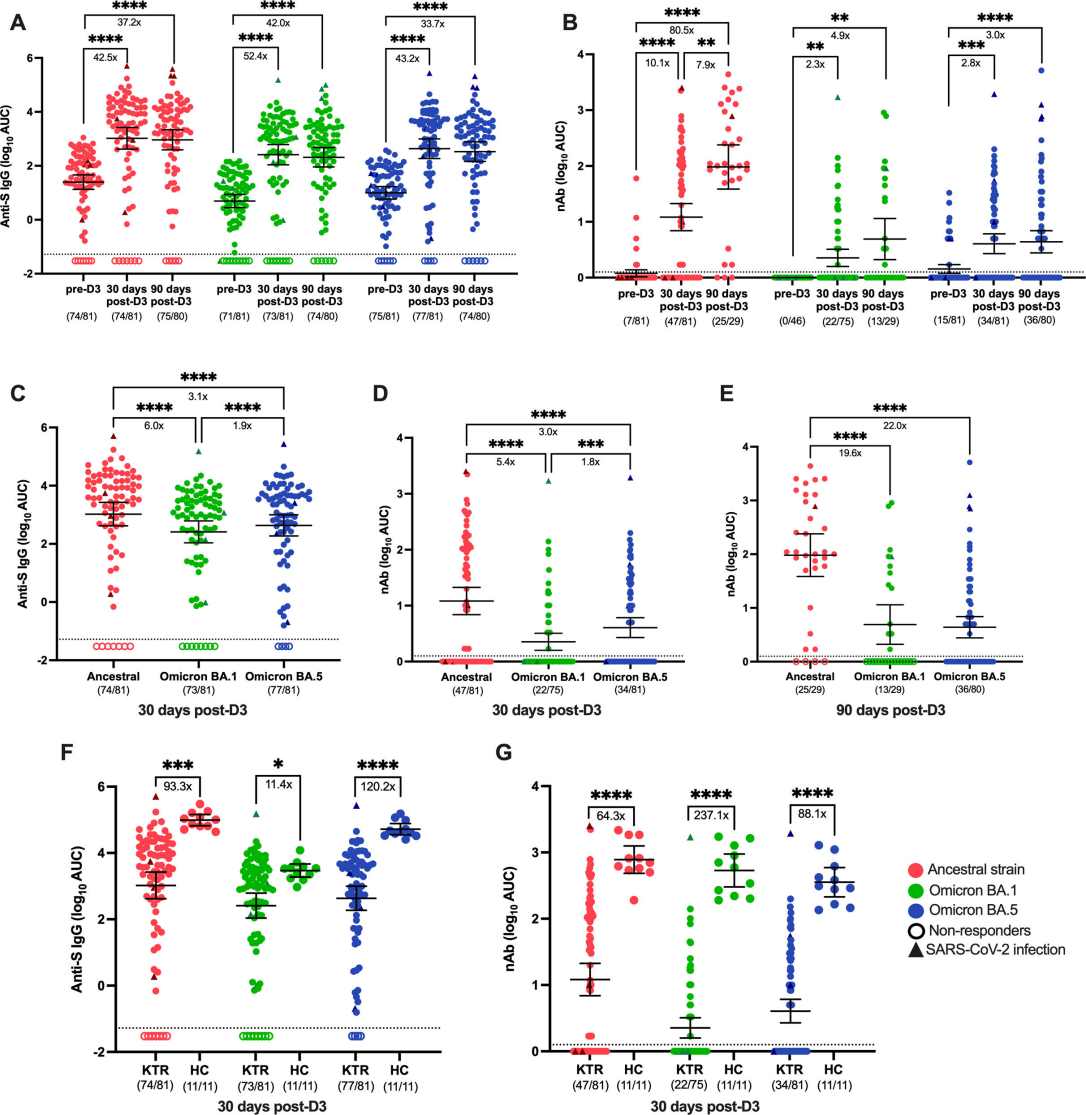
Serum IgG and nAb responses to SARS-CoV-2 ancestral and Omicron variants in KTRs in comparison to HCs. Total SARS-CoV-2 Spike (S)-specific IgG (**A**) and neutralizing antibody (nAb) (**B**) against the ancestral strain (red), Omicron BA.1 (green), and Omicron BA.5 (blue) variants measured in KTRs (*n* = 81) or HCs (*n* = 11) who were previously vaccinated with the initial two-dose mRNA vaccine series prior to dose 3 (pre-D3), 30 days post-dose 3 (30 days post-D3), and 90 days post-dose 3 (90 days post-D3). Total anti-S IgG (**C**) and nAb levels (**D**) at 30 days post-D3 and nAb levels at 90 days post-D3 (**E**) were compared between the ancestral strain, Omicron BA.1, and Omicron BA.5 variants. Total anti-S IgG (**F**) and nAb (**G**) levels against ancestral strain, Omicron BA.1, and Omicron BA.5 variants were compared between KTR and HCs at 30 days post-D3. Dotted lines indicate the limit of detection (LOD), which is −1.52 for the anti-S IgG ELISA assay (**A, C, F**) and 0.17 for the nAb assay (**B, D, E, G**). Open circles represent non-responders with negative serological responses that fall below the LOD value. Solid triangles represent patients with a confirmed SARS-CoV-2 infection during the course of the study. The mean ± 95% CI are shown in each panel. Significance is tested using one-way repeated measures ANOVA (**A–E**), and unpaired t-tests (**F, G**). *P < 0.05, **P < 0.01, ***P < 0.001, and ****P < 0.0001. Fold changes (X) are labeled below the significance lines. The number of positive samples out of the total number of samples tested is indicated in parentheses.

The magnitude and breadth of pre- and post-dose 3 (D3) vaccination antibody responses to SARS-CoV-2 variants were assessed in KTRs. At 30 days post-D3, IgG binding to the S protein showed a substantial increase for the ancestral strain, with 91% of individuals having increased antibody levels; the average increase in IgG binding antibodies was 42.5-fold (Fig. 1A). At 90 days post-D3, 94% of individuals had higher antibody levels compared with baseline, and the average increase is 37.2-fold. For the Omicron BA.1 variant, 91% of individuals had detectable antibodies, which corresponded with a 52.4-fold increase in antibody titers at 30 days post-D3. By 90 days post-D3, 93% still had detectable antibodies, and levels were increased by 42.0-fold compared with pre-D3. For the Omicron BA.5 variant, 95% of individuals had detectable antibodies at 30 days post-D3, representing a 43.2-fold increase from pre-D3. The number of antibody positive individuals at 90 days post-D3 was 93%, and this population had a 33.7-fold increase in antibody levels compared with pre-D3. Anti-S responses were maintained through 90 days post-D3 for all three variants.

The number of individuals with detectable live virus neutralizing antibody (nAb) responses against the ancestral strain increased significantly from pre-D3 to 30 days post-D3 (9% to 58%) and remained elevated at 90 days post-D3 (86%). The nAb titers increased 10.1-fold at 30 days post-D3 and were 7.9-fold higher at 90 days post-D3 compared to pre-D3 (Fig. 1B). For BA.1, the number of individuals with nAb titers increased to 29% at 30 days post-D3 and 45% at 90 days post-D3. The BA.1 nAb titers increased 2.3-fold at 30 days post-D3 and were 4.9-fold higher at 90 days post-D3 compared with pre-D3 (Fig. 1B). BA.5 exhibited a similar pattern. The number of individuals with nAb titers increased to 42% at 30 days post-D3 and 45% at 90 days post D-3. The BA.5 nAb titers increased 2.8-fold at 30 days post-D3 and were 3.0-fold higher at 90 days post-D3 compared with pre-D3 (Fig. 1B). These data indicate that a subset of KTRs receiving a third dose of ancestral Spike (S) vaccine were able to mount binding and nAb responses to divergent Omicron variants.

Generally, we found that the magnitude of the anti-S responses increased most rapidly during the first 30 days after D3 vaccination, while the nAb responses to the ancestral strain continually increased until 90 days post-D3 (Fig. 1A and B), though limited plasma volumes did impact the number of samples that were analyzed against some variants. Although a third-dose mRNA vaccine induced responses to all three strains of SARS-CoV-2, responses to the Omicron BA.1 variant (6.0-fold decrease in anti-S IgG; 5.4-fold decrease in nAb; *P* < 0.0001) and Omicron BA.5 variant (1.9-fold decrease in anti-S IgG; *P* < 0.0001; 1.8-fold decrease in nAb; *P* < 0.0.1) were significantly lower when compared with the ancestral strain 30 days after D3 (Fig. 1C and D). The nAb responses to Omicron BA.1 (19.6-fold decrease; *P* < 0.0001) and Omicron BA.5 (22.0-fold decrease; *P* < 0.0001) were also significantly lower than ancestral strain 90 days post-D3 (Fig. 1E).

We next compared serological responses between vaccinated KTRs and HCs at 30 days post-D3 to determine if there were differences in vaccine-induced immunity between immunocompromised KTRs and immunocompetent HCs. For binding antibodies, the number of responders was similar between HC and KTRs against ancestral, BA.1, and BA.5, when compared with HC. After receipt of the third COVID-19 mRNA vaccine, all HCs had detectable anti-S IgG responses against the three Spike variants, and the percentage of responders in the KTRs was also high (91% to ancestral Spike, 90% to BA.1, and 95% to BA.5) (Fig. 1F). However, KTRs had consistently lower total antibody recognizing ancestral Spike (64.3-fold), BA.1 (11.4-fold), and BA.5 (120.2-fold) when compared with HCs (Fig. 1F).

Similarly, all HCs had detectable nAb responses post-D3 against the ancestral, BA.1, and BA.5 variants, but these numbers were reduced in KTRs with the percentage of responders being 58% against the ancestral strain, 29% against BA.1, and 42% against BA.5 (Fig. 1G). As compared with HCs, KTRs also showed significant reductions in nAb titers, with reductions of 64.3-fold, 237.1-fold, and 88.1-fold for ancestral, BA.1, and BA.5, respectively (Fig. 1G). Over half of KTRs showed non-detectable nAb titers against both the ancestral virus and Omicron variants, but HCs demonstrated a 100% response rate at 30 days post-D3 (Fig. 1G). Taken together, these data suggested that KTRs mount improved antibody responses to a third dose of mRNA vaccine, but the titers remained lower, and a lower proportion of KTRs mounted detectable responses.

### KTRs mount lower SARS-CoV-2 Spike-specific IgG subclasses than healthy controls HCs after a third dose of vaccine

**Fig 2:**
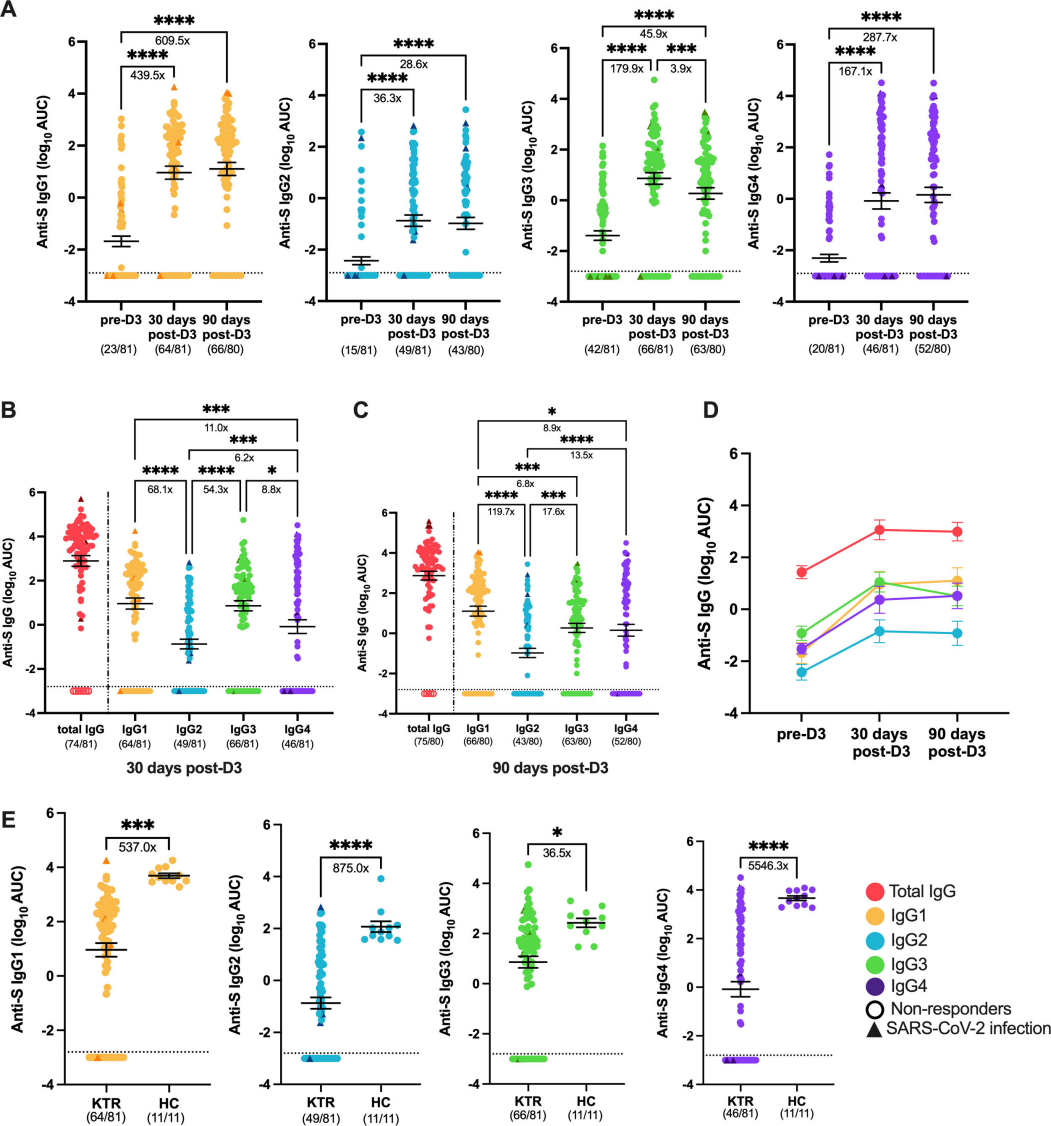
Serum IgG subclass profile to SARS-CoV-2 ancestral strain in KTRs. SARS-CoV-2 Spike (S)-specific IgG subclasses (IgG1-4): IgG1 (yellow), IgG2 (blue), IgG3 (green), and IgG4 (purple) against ancestral strain were measured in KTRs (*n* = 81) prior to dose 3 (pre-D3), 30 days post-dose 3 (30 days post-D3), and 90 days post-dose 3 (90 days post-D3) (**A**). Anti-S IgG subclasses antibody levels against SARS-CoV-2 ancestral strain were compared at 30 days post-D3 (**B**) and 90 days post-D3 (**C**) in KTRs with anti-S total IgG (red) as a reference on the left of both panels. A summary panel of anti-S IgG subclass-specific antibody levels against ancestral strain is shown and connected by lines to show the changes of serological responses in IgG subclasses from pre-D3, 30 days post-D3, to 90 days post-D3 (**D**). Comparison of anti-S IgG subclass-specific antibody levels against ancestral strain was made between KTRs and healthy controls (HCs) (*n* = 11) at 30 days post-D3 (**E**). Dotted lines indicate the limit of detection (LOD), which is −3.00 for the subclass-specific IgG ELISA assay. Open circles represent non-responders with negative serological responses that fall below the LOD value. olid triangles represent patients with a confirmed SARS-CoV-2 infection during the course of the study. The mean ± 95% CI are shown in each panel. Significance is tested using mixed-effects model (**A**), one-way repeated measures ANOVA (**B, C**), and unpaired *t*-test (**E**). *P < 0.05, **P < 0.01, ***P < 0.001, and ****P < 0.0001. Fold changes (X) are labeled below the significance lines. The number of positive samples out of the total number of samples tested is indicated in parentheses.

The abundance of subclass-specific IgG antibodies to SARS-CoV-2 ancestral S was also assessed in KTRs after D3. There were differences in the proportion of individuals who generated subclass specific antibody responses post-D3. Analyses of the proportion of participants with detectable IgG subclass responses illustrated that anti-ancestral S IgG1 had 79% responders and 439.5-fold increase to 30 days post-D3 and 83% responders and a 609.5-fold increase to 90 days post-D3. The anti-ancestral S IgG2 showed 60% responders and a 36.3-fold increase to 30 days post-D3 and 54% responders and a 28.6-fold increase to 90 days post-D3. The anti-ancestral S IgG3 had 81% responders and a 179.9-fold increase to 30 days post-D3 and 79% responders and a 45.9-fold increase to 90 days post-D3. The anti-ancestral S IgG4 had 57% responders and a 167.1-fold increase to 30 days post-D3 and 65% responders and a 287.7-fold increase to 90 days post-D3. The IgG1, IgG3, and IgG4 responses were the strongest in this cohort, with IgG2 responses showing the lowest increase at days 30 and 90 post-D3 (Fig. 2A). There was a significant decrease in the abundance of IgG3 between 30 days to 90 days post-D3 (3.9-fold decrease). At 30 days post-D3, IgG1 (68.1-fold increase than IgG2; 11.0-fold increase than IgG4) and IgG3 (54.3-fold increase than IgG2; 8.8-fold increase than IgG4) titers were greater than titers of IgG2 and IgG4 (Fig. 2B). The abundance of IgG subclasses changed at 90 days post-D3 with comparable IgG3 and IgG4 antibody levels (Fig. 2C). Overall, among KTRs, there was an increase in all IgG subclasses post-D3, with IgG1 as the dominant subclass in KTRs after D3 (Fig. 2D).

We next compared IgG subclass responses between KTRs and HCs 30 days post-D3 to determine if there were differences in vaccine-induced IgG subclasses between immunocompromised KTRs and immunocompetent individuals. As compared with HCs, KTRs had lower IgG1 (537.0-fold decrease), IgG2 (875.0-fold decrease), IgG3 (36.5-fold decrease), and IgG4 (5546.3-fold decrease) responses (Fig. 2E). These findings suggested that KTRs mounted significantly lower anti-S IgG responses across all subclasses than HCs after a third dose of the mRNA vaccine.

## DISCUSSION

This study demonstrated that while a third dose of mRNA COVID-19 vaccine elicited anti-S IgG and nAb responses against Omicron variants in KTRs, these responses were consistently lower and highly heterogenous compared with HCs. Previous literature has demonstrated a correlation between antigen-specific antibodies and neutralizing antibodies, which are both deemed as protective correlates for SARS-CoV-2 vaccination and COVID-19 breakthrough diseases in immunocompetent individuals (45). In this study, robust neutralization was not observed in KTRs despite high anti-S titers; however, KTRs still had a significantly reduced level of spike-specific antibodies compared with HCs. The level of clinical protection correlating with antibody activity was not fully studied here due to the lack of reported clinical breakthrough infections. It is not obvious if either anti-S or nAb antibody response provide adequate protection to immunocompromised populations from symptomatic or severe COVID-19. Notably, even after a third dose, substantial variability in serological responses was observed in KTRs, reflecting the influence of factors, such as immunosuppressive medication history, comorbidities, and previous exposure to COVID-19.

The data presented in this study provide evidence that a third dose of mRNA SARS-CoV-2 vaccine increases serological responses for some KTRs against SARS-CoV-2 ancestral versus the Omicron BA.1 and BA.5 variants. More than half of the KTRs had no detectable nAb titers against Omicron variants, suggesting that this could be attributed to a more potent state of immunosuppression or more complete antigenic imprinting with reduced recognition of the VOC that was similar but not identical to the ancestral virus. Importantly, KTRs have significantly decreased antibody responses against all three variants compared with HCs after receipt of a third-dose SARS-CoV-2 vaccine. These differences indicate that COVID-19 vaccination results in a highly variable level of protection among different KTRs due to inherent immunological differences at baseline. Moreover, the subclass-specific anti-S IgG antibody profile against the SARS-CoV-2 ancestral strain predominantly consisted of IgG1 and IgG3 at 30 days after D3, but there was a significant decrease in IgG3 after 90 days post-D3. It is probable that the reduction in IgG3 is a result of its shorter half-life, which serves to restrict the occurrence of excessive inflammatory responses. It is also important to note that IgG3 antibodies are known for their effectiveness in removing immune complexes, and that a reduction of IgG3 could greatly impact the ability to engage with the complement system.

Several limitations of this study should be acknowledged. The HC comparison group was younger than the KTR cohort, which might contribute to the observed differences in antibody responses. Additionally, not all clinical specimens were available for every variant or antibody subtype, potentially skewing the results. Although nAbs are important correlates of protection, their levels alone may not provide a complete picture of immune defense, particularly in the immunocompromised. The role of non-neutralizing antibodies and Fc-mediated functions, such as antibody-dependent cellular cytotoxicity (ADCC) and complement activation, remains underexplored but could play a critical role in modulating COVID-19 disease severity in these populations. In summary, the study informs us that SARS-CoV-2 vaccination and boosting may not be a fully effective strategy in KTRs, and that consideration should be given to different formulations and doses of COVID-19 vaccines in people with severely compromised immune systems, as more frequent vaccinations may not significantly increase the non-responding group, particularly when it comes to neutralizing antibodies.
